# Current investigation of neurocognitive functioning in preschool children with cancer: A cross-sectional study from western China

**DOI:** 10.1371/journal.pone.0312536

**Published:** 2024-11-11

**Authors:** Zefang Chen, Lifang Xu, Lin Mo

**Affiliations:** 1 Department of Nursing Children’s Hospital of Chongqing Medical University, Chongqing, China; 2 National Clinical Research Center for Child Health and Disorders, Ministry of Education Key Laboratory of Child Development and Disorders, Chongqing, China; 3 Chongqing Key Laboratory of Child Neurodevelopment and Cognitive Disorders, Chongqing, China; 4 Department of Outpatient, Children’s Hospital of Chongqing Medical University, Chongqing, China; University of Padua, ITALY

## Abstract

**Background and aims:**

Cancer and its treatments may cause neurocognitive impairments in preschool children, but there is limited research on the neurocognitive outcomes of this population. This study, which assessed the neurocognitive function of preschool children with cancer and analyzed various influencing factors of neurocognitive functioning, is of significant importance. We aimed to investigate neurocognitive function and related risk factors in preschool children with cancer to inform preventive and intervention strategies.

**Methods:**

From September 2023 to May 2024, we recruited 100 preschool children with cancer. The Chinese version of the Ages & Stages Questionnaires, the Spence Preschool Anxiety Scale Chinese Version, and the Sleep Disturbance Scale for Children were used to collected data. Binary logistic stepwise regression analysis was used to explore the influencing factors of neurocognitive function in preschool children with cancer.

**Results:**

49% of the preschool children with cancer had abnormalities in at least one neurocognitive dimension. The majority of children had abnormalities in gross motor dimension, accounting for 30%, which was related to age and frequency of participation in neurocognitive activities. Communication dimension was related to father’s education level, dietary habit, and frequency of participation in activities. Fine motor dimension was associated with age, sex, and father’s education level. Problem-solving dimension was associated with age and dietary habit. Personal-social dimension was related to age and radiotherapy.

**Conclusions:**

Nearly half of preschool children with cancer experienced neurocognitive impairment. The Chinese version of the Ages & Stages Questionnaires is a simple and effective tool for screening children with possible neurocognitive impairment. It was found that children’s neurocognitive function was significantly influenced by family environment, dietary habit, cognitive activities, and cancer treatment. Therefore, it is recommended to strengthen family and social support, and to formulate personalized intervention such as cognitive therapy and dietary adjustment based on children’s age and family background, which are important for promoting neurocognitive recovery.

## 1. Introduction

Cancer is one of the most common diseases, affecting the health of children and adolescents, with approximately 400,000 children aged 0 to 19 years old worldwide annually [1]. In China, about 40,000 children are diagnosed with cancer every year, bringing a heavy burden to their families and society [2]. With the rapid development of medical technology, the survival rate of cancer children has been improved year by year. In developed countries, the five-year survival rate of children with cancer has exceeded 80% [3, 4]. However, many children still face various side effects of the disease, with the most common being neurocognitive impairment, which may have a profound impact on the long-term development of children.

Neurocognitive impairment caused by different types of cancer and their treatments is a common complication [5]. It mainly manifestas significant loss or deficiency of neurocognitive functions, such as memory, attention, language expression, executive ability, and problem-solving speed [6–8]. Early screening helps detect and prevent neurocognitive deficits in children. However, due to the limited ability of preschool children to express themselves clearly, the insufficient attention from parents and clinical doctors to these issues by parents and clinicians, and the fact that preschool children are in a critical period of rapid brain development, the incidence of neurocognitive impairment in children with cancer is as high as 35% to 60% [9]. Neurocognitive delays may lead to an increasing risk of memory impairment or early-onset dementia in children over time [10]. Especially for preschool children, neurocognitive deficits during this period may have a lasting impact on their learning ability and future education [11–13]. Due to the highly active brain development in preschool children, the impact of cancer and related treatments on neurocognitive function may be more severe compared to older children [14]. Therefore, early identification and management of neurocognitive impairment are critical to optimize the long-term development of these young patients.

The risk factors for neurocognitive impairment in children with cancer are related to physiology, psychology, and disease treatment [15–17]. Related studies have shown [18] that cranial radiotherapy and intrathecal chemotherapy are high risk factors for neurocognitive impairment in children at a young age. Moleski [19] has demonstrated that children receiving intrathecal chemotherapy are at risk of long-term changes in tissue morphology and brain function. Lin [20] has found that changes in nutritional status may cause changes in brain-related functions, and an adequate diet helps the development of brain function. Hong et al. [21] found that 79.52% of 166 children and adolescents with cancer had sleep disorders and were related to neuropsychological function. Reuter et al. [17] have demonstrated that neurotransmitters are closely related to psychological aspects, such as pentraxinergic, which is essential for children’s cognitive development. Albee’s [21] study has emphasized that a harmonious family atmosphere is beneficial for children with the disease to acquire better social skills, while poor family relationships may exacerbate emotional disorders, leading to negative psychology and negative emotions and increasing the risk of cognitive impairment. Unfortunately, current studies on neurocognitive function focus on risk factors at the individual level, emphasizing changes in clinical indicators. During the critical growth period of preschool years, it is essential to conduct early comprehensive screening at multiple levels, including physiological, psychological, and disease treatments, to move the prevention of neurocognitive impairment forward, which can promote brain development, reduce neurological damage to varying degrees, and prevent or reduce cognitive impairment.

Through a cross-sectional survey conducted in a tertiary children’s hospital in western China, we aimed to explore the neurocognitive function status of preschool children with cancer and to comprehensive analyze the influencing factors for neurocognitive impairment in children with cancer. We expect to provide a basis for clinical practitioners and related researchers to develop individualized interventions to effectively prevent or reduce the occurrence of neurocognitive impairment in children with cancer.

## 2. Materials and methods

### 2.1 Study participants

From September 2023 to May 2024, convenience sampling method was used to recruited, preschool children with cancer hospitalized at children’s hospital of Chongqing medical university as participants. Due to the convenience sampling method, only children whose parents agreed to participate in this study were included in the study. Most children hospitalized at this hospital were accompanied by their parents.

Inclusion criteria: ① cancer (including but not limited to central nervous system tumors, hematological tumors, etc.) confirmed by clinical pathological diagnosis according to established guidelines for the diagnosis and treatment of cancer in children;② between the ages of 2 years six months to 5 years six months; ③ children and their caregivers did not have any psychiatric severe illnesses or recent significant psychological stresses; ④ informed consent and voluntary participation, and had the ability to communicate verbally.

Exclusion criteria:① severe physical disorder;② caregivers had hearing impairment or cognitive impairment.

## 2.1.1 Ethics approval and consent to participate

The study followed the Declaration of Helsinki and was approved by the Ethics Committee (full name: Institutional Review Board of Children’s Hospital of Chongqing Medical University) (reference number [2023 (year) No.270]) to the Department of Scientific Research, Children’s Hospital of Chongqing Medical University, China. We carried out all methods according to the relevant guidelines and regulations. This study was carried out in compliance with the STROBE Statement. All included patients gave their oral and written informed consent. The study was voluntary, and patients will permit this material to appear in academic journals and associated publications. In addition, data adhered to the principle of confidentiality, private information such as the child’s name was not disclosed, the primary caregiver signed an informed consent form, and the survey was completed anonymously.

### 2.2 Outcomes and measurements

#### 2.2.1 General information

The general information questionnaire was developed through a review of relevant literature, discussion within the research team, and correspondence with oncologists. The general information included socio demographic data, such as the sex of the child, age of the child, number of hospitalizations, age at initial diagnosis, educational level of the primary caregiver, residence, child disposition, and parenting style (authoritarian, autocratic, and indulgent, with corresponding explanations provided for each option to allow respondents to choose based on their actual situation), and disease and treatment-related data, such as surgical interventions received, radiotherapy and chemotherapy, use of chemotherapeutic agents, and laboratory test results.

## 2.2.2 Neurocognitive function

We used the Chinese version of Ages & Stages Questionnaires (ASQ-3) to assess neurocognitive function in preschool children with cancer, which was developed by Bianet al. [22]. ASQ-3 is used to assess the cognitive developmental behaviors in children aged 36 to 66 months. The ASQ-3 is often used to screen children’s cognitive function because it is a recommended scale by the United National International Children’s Emergency Fund. Studies have shown that the Cronbach’s coefficient of the Chinese version of the ASQ-3 is 0.8, and the sensitivity and specificity of the ASQ-3 are 87.50% and 84.48%, respectively.

This scale has five dimension, including communication, gross motor, fine motor, problem-solving, and personal-social. Each dimension consists of 6 items with three options: “Yes” indicating that the child can complete the item (10 points), “Sometimes” indicating that the child can partially complete the item (5 points), and “No” indicating that the child failed to complete the item (0 points). When we compared children’s cognitive function in this study with the norms of urban children in Chinese mainland, the screening results of each domain were divided into three groups. If the dimension score was close to or below the threshold, child would be defined as suspected neurocognitive impairment. Otherwise, child would be defined as functioning normally.

## 2.2.3 Anxiety

We used the Chinese version of the Spence Preschool Anxiety Scale (PAS) [23] to assess the anxiety in preschool children with cancer. The PAS consists of 28 items and 5 dimensions: separation anxiety, fear of bodily harm, social phobia, obsessive-compulsive disorder, and generalized anxiety, as well as five items for post-traumatic stress disorder. Disorder (PTSD) and five post-traumatic stress disorder (PTSD) items are 33 in total. A 5-point scale (0–4) was used, with the higher the score, the more severe the anxiety. The Cronbach’s coefficient of the PAS scale is 0.87, indicating good reliability among children in the China.

## 2.2.4 Sleep disorders

The Sleep Disturbance Scale for Children (SDSC) was used in this study to assess sleep disorders in preschool children with cancer. There are 26 items in total, rated using the Likert 5-point scale according to the frequency of occurrence. The scores are as follows: 1 point = never, 2 points = 1–2 times per month, 3 points = 1–2 times per week, 4 points = 3–5 times per week, and 5 points = always. The total score of each part is the sum of the scores, and a score greater than 39 can be considered a sleep disorder. The SDSC scale can reflect some of the most common sleep disorders affecting children. The Cronbach’s coefficient of the SDSC scale is 0.79.

### 2.3 Data collection

Trained healthcare professionals collected data and performed the ASQ screening for this study in a hospital setting. After providing informed consent from parents, the healthcare professionals guided the primary caregivers children to answer the questions on the scale appropriately during the screening process. Scores were assigned according to the screening criteria. The test duration was 15 minutes, and all the questions were asked without hints or demonstrations, in accordance with the questionnaire guidelines.

### 2.4 Statistical methods

We carried out the statistical analysis using SPSS26.0 software. For measurement data with normal distribution, it was expressed as *x*±*s*, and the comparison between groups was conducted by independent samples T-test. Multiple groups were compared by one-way analysis of variance (ANOVA). For measurement data with non-normal distribution, it was described as M (Q_25_-Q_75_), and the Mann-Whitney U test was used to conduct the comparison between groups. The Kruskal-Wallis H test was used to compare multiple groups. Count data was presented as case numbers and percentages (%), and the comparisons between groups were performed using the Chi-square test. The Wilcoxon rank sum test was used to test for multi-class variables across groups. Multiple factor analysis was conducted using binary logistic stepwise regression analysis to test the related factors of neurocognitive impairment in children with cancer. To ensure the robustness of our regression model, we checked for multicollinearity among the predictors using Variance Inflation Factors (VIFs). All VIF values were found to be below the threshold of 10 (more conservatively, below 5), indicating that multicollinearity was not a significant issue in our model. The *P*<0.05 indicated significant differences.

## 3 Results

### 3.1 The basic information of children with cancer

As shown in Table 1, a total of 100 preschool children aged 36–66 months with cancer were screened, including 50 males (50%) and 50 females (50%), with a sex ratio of 1:1. The mean age was (4.80±1.33) years old, with males (4.76±1.37) years old and females (4.87±1.29) years old.

**Table 1:** Demographic characteristics of children with cancer and their caregivers (n = 100).

Variables	M (Q_25_, Q_75_)/n(%)	Variables	M (Q_25_, Q_75_)/n(%)
**Times of hospitalisations**	8.00 (3.00, 15.00)	**Mother’s education level**	
**Age at first diagnosis**	3.25 (2.23, 4.27)	Primary school and below	**11 (11.00)**
**Duration of disease**	1.08 (0.40, 2.35)	Junior High School	**29 (29.00)**
**Age**		High School	**24 (24.00)**
36–41 months	15 (15.00)	University and above	**36 (36.00)**
42–47 months	12 (12.00)	**Primary caregiver**	
48–53 months	9 (9.00)	parent	**82 (82.00)**
54–59 months	17 (17.00)	non-parent	**18 (18.00)**
≥60 months	47 (47.00)	**Residence**	
**Sex**		Urban	**57 (57.00)**
Male	50 (50.00)	Rural	**43 (43.00)**
Female	50 (50.00)	**Child disposition**	
**Type of cancer**		Extroversion	**49 (49.00)**
neurological cancer	2 (2.00)	Centralization	**38 (38.00)**
hematologic cancer	14 (14.00)	ntrovert	**13 (13.00)**
Other cancer	84 (84.00)	**Dietary habit**	
**Surgery**		balanced diet	**81 (81.00)**
no	24 (24.00)	meat-based diet	**9 (9.00)**
yes	76 (76.00)	vegetarianism diet	**10 (10.00)**
**Radiotherapy**		**physical exercise**	
no	76 (76.00)	Never	**6 (6.00)**
yes	24 (24.00)	Regular	**22 (22.00)**
**Chemotherapy**		Irregular	**72 (72.00)**
no	5 (5.00)	**Frequency of cognitive activity**	
yes	95 (95.00)	Never	**18 (18.00)**
**Intrathecal chemotherapy**		Regular	**17 (17.00)**
no	27 (27.00)	Irregular	**65 (65.00)**
yes	73 (73.00)	**Daily time spent using the Internet**	
**Parenting style**		<2 hours	**51 (51.00)**
Authoritative type	74 (74.00)	2–6 hours	**44 (44.00)**
Autocratic type	8 (8.00)	>6 hours	**5 (5.00)**
Doting type	18 (18.00)	**Comorbidity**	
**Income (RMB)**		no	**90 (90.00)**
<3000	27 (27.00)	yes	**10 (10.00)**
3000–5000	41 (41.00)	**Sleep duration**	
5000–10000	20 (20.00)	9–11 hours.	**58 (58.00)**
>10000	12 (12.00)	8–9 hours.	**36 (36.00)**
**Father’s education level**		7–8 hours.	**5 (5.00)**
Primary school and below	7 (7.00)	5–7 hours.	**1 (1.00)**
Junior High School	33 (33.00)		
High School	22 (22.00)		
University and above	38 (38.00)		

### 3.2 Neurocognitive function

The 49 out of 100 screened children scored below the cut-off value in at least one dimension and were suspected to hadneurocognitive impairment, giving an overall neurocognitive function abnormality rate of 49%. As shown in Table 2, the screening results for the different dimensions revealed that the highest percentage of below-boundary values (30%) was found in gross motor, and the highest percentage of near-boundary values (23.21%) was found in problem-solving dimension.

**Table 2:** Neurocognitive function scores in 100 preschool children with cancer [n(%)].

Dimensions	Scores ($\bar{x}\pm s$)	Normal	-1<standard deviation ≥-2	2< standard deviation
Communication	53.85±8.90	86 (86)	12 (12)	2 (2)
Gross motor	49.50±11.80	70 (70)	17 (17)	13 (13)
Fine motor	44.35±15.05	77 (77)	11 (11)	12 (12)
Problem Solving	51.45±11.67	81 (81)	11 (11)	8 (8)
Personal-Social	51.60±10.80	77 (77)	13 (13)	10 (10)

### 3.3 Univariate analysis of neurocognitive impairment

By using VIFs for all the variables in Table 3, it was found that all VIF values were found to be below the threshold of 10, indicating that there was no multicollinearity between the variables. The univariate analysis for each dimension is summarized in Table 4, and the analysis taken for the different types of variables are noted at the end of the table.

**Table 3:** VIFs results for all the variables.

Variables	VIF	Variables	VIF
Times of hospitalisations	1.380	Mother’s education level	3.487
Age at first diagnosis	2.330	Primary caregiver	1.238
Duration of disease	2.455	Residency	2.292
Sex	1.157	Disposition	1.171
Type of cancer	1.716	dietary habit	1.253
Surgery	2.145	physical exercise	1.440
Radiotherapy	1.430	cognitive activity	1.362
Chemotherapy	1.274	Daily time spent using the Internet	1.347
Intrathecal chemotherapy	2.077	Parenting style	1.370
Father’s education level	3.836	Income	1.828

**Table 4:** Univariate analysis of neurocognitive impairment in preschool children with cancer (n = 100).

Variables	Communication [M (Q25, Q75)/n(%)]		Z/χ^2^	P	Gross motor [M (Q25, Q75)/n(%)		Z/χ^2^	P	Fine motor [M (Q25, Q75)/n(%)		Z/χ^2^	P	Problem Solving [M (Q25, Q75)/n(%)]		Z/χ^2^	P	Personal-Social [M (Q25, Q75)/n(%)]		Z/χ^2^	P
	0 (n = 86)	1 (n = 14)			0 (n = 86)	1 (n = 14)			0 (n = 86)	1 (n = 14)			0 (n = 86)	1 (n = 14)			0 (n = 86)	1 (n = 14)		
**Age at first diagnosis**	**3.38 (2.35, 4.33)**	**2.21 (0.83, 3.52)**	**-2.01**	**0.044**	**3.42 (2.25, 4.48)**	**3.00 (2.17, 4.17)**	**-0.77**	**0.441**	**3.33 (2.25, 4.33)**	**3.08 (2.17, 4.21)**	**-0.43**	**0.667**	**3.42 (2.25, 4.33)**	**2.58 (2.17, 4.04)**	**-0.97**	**0.331**	**3.33 (2.25, 4.33)**	**3.08 (2.12, 4.25)**	**-0.46**	**0.643**
**Duration of disease**	**1.04 (0.35, 2.40)**	**1.25 (0.54, 1.92)**	**-0.45**	**0.655**	**1.21 (0.42, 2.90)**	**0.88 (0.27, 1.56)**	**-1.40**	**0.161**	**1.25 (0.42, 2.92)**	**0.83 (0.25, 1.25)**	**-2.12**	**0.034**	**1.17 (0.42, 2.83)**	**0.67 (0.21, 1.33)**	**-1.88**	**0.060**	**1.25 (0.42, 3.00)**	**0.67 (0.21, 1.33)**	**-2.39**	**0.017**
**PAS**	**31.00 (19.25, 40.00)**	**34.00 (22.75, 37.75)**	**-0.16**	**0.874**	**30.50 (20.00, 39.75)**	**33.00 (18.75, 39.00)**	**-0.07**	**0.946**	**31.00 (20.00, 40.00)**	**26.00 (20.50, 34.50)**	**-1.46**	**0.144**	**31.00 (20.00, 39.00)**	**24.00 (19.00, 39.50)**	**-0.59**	**0.556**	**31.00 (19.00, 39.00)**	**32.00 (21.00, 39.00)**	**-0.16**	**0.873**
**Age group**			**-**	**0.153**			**-**	**0.017**			**-**	**0.143**			**-**	**0.087**			**-**	**0.024**
36–41 months	**11 (12.79)**	**4 (28.57)**			**9 (12.86)**	**6 (20.00)**			**8 (10.39)**	**7 (30.43)**			**9 (11.11)**	**6 (31.58)**			**7 (9.09)**	**8 (34.78)**		
42–47 months	**11 (12.79)**	**1 (7.14)**			**8 (11.43)**	**4 (13.33)**			**10 (12.99)**	**2 (8.70)**			**10 (12.35)**	**2 (10.53)**			**11 (14.29)**	**1 (4.35)**		
48–53 months	**6 (6.98)**	**3 (21.43)**			**4 (5.71)**	**5 (16.67)**			**6 (7.79)**	**3 (13.04)**			**6 (7.41)**	**3 (15.79)**			**6 (7.79)**	**3 (13.04)**		
54–59 months	**15 (17.44)**	**2 (14.29)**			**9 (12.86)**	**8 (26.67)**			**15 (19.48)**	**2 (8.70)**			**14 (17.28)**	**3 (15.79)**			**13 (16.88)**	**4 (17.39)**		
≥60 months	**43 (50.00)**	**4 (28.57)**			**40 (57.14)**	**7 (23.33)**			**38 (49.35)**	**9 (39.13)**			**42 (51.85)**	**5 (26.32)**			**40 (51.95)**	**7 (30.43)**		
**Sex**			**5.32**	**0.021**			**0.19**	**0.663**			**6.83**	**0.009**			**0.58**	**0.444**			**4.57**	**0.032**
Male	**39 (45.35)**	**11 (78.57)**			**36 (51.43)**	**14 (46.67)**			**33 (42.86)**	**17 (73.91)**			**39 (48.15)**	**11 (57.89)**			**34 (44.16)**	**16 (69.57)**		
Female	**47 (54.65)**	**3 (21.43)**			**34 (48.57)**	**16 (53.33)**			**44 (57.14)**	**6 (26.09)**			**42 (51.85)**	**8 (42.11)**			**43 (55.84)**	**7 (30.43)**		
**Radiotherapy**			**0.59**	**0.442**			**0.17**	**0.683**			**0.68**	**0.410**			**0.00**	**1.000**			**3.75**	**0.053**
no	**67 (77.91)**	**9 (64.29)**			**54 (77.14)**	**22 (73.33)**			**60 (77.92)**	**16 (69.57)**			**62 (76.54)**	**14 (73.68)**			**62 (80.52)**	**14 (60.87)**		
yes	**19 (22.09)**	**5 (35.71)**			**16 (22.86)**	**8 (26.67)**			**17 (22.08)**	**7 (30.43)**			**19 (23.46)**	**5 (26.32)**			**15 (19.48)**	**9 (39.13)**		
**Father’s education level**			**-**	**0.012**			**-**	**0.409**			**8.51**	**0.037**			**-**	**0.005**			**2.64**	**0.451**
Primary school	**4 (4.65)**	**3 (21.43)**			**5 (7.14)**	**2 (6.67)**			**3 (3.90)**	**4 (17.39)**			**5 (6.17)**	**2 (10.53)**			**4 (5.19)**	**3 (13.04)**		
Junior High School	**27 (31.40)**	**6 (42.86)**			**20 (28.57)**	**13 (43.33)**			**24 (31.17)**	**9 (39.13)**			**27 (33.33)**	**6 (31.58)**			**24 (31.17)**	**9 (39.13)**		
High School	**18 (20.93)**	**4 (28.57)**			**15 (21.43)**	**7 (23.33)**			**16 (20.78)**	**6 (26.09)**			**13 (16.05)**	**9 (47.37)**			**18 (23.38)**	**4 (17.39)**		
University and above	**37 (43.02)**	**1 (7.14)**			**30 (42.86)**	**8 (26.67)**			**34 (44.16)**	**4 (17.39)**			**36 (44.44)**	**2 (10.53)**			**31 (40.26)**	**7 (30.43)**		
**Residence**			**3.01**	**0.083**			**5.05**	**0.025**			**8.60**	**0.003**			**3.89**	**0.049**			**3.89**	**0.049**
urban area	**52 (60.47)**	**5 (35.71)**			**45 (64.29)**	**12 (40.00)**			**50 (64.94)**	**7 (30.43)**			**50 (61.73)**	**7 (36.84)**			**48 (62.34)**	**9 (39.13)**		
Rural	**34 (39.53)**	**9 (64.29)**			**25 (35.71)**	**18 (60.00)**			**27 (35.06)**	**16 (69.57)**			**31 (38.27)**	**12 (63.16)**			**29 (37.66)**	**14 (60.87)**		
**Dietary habit**			**-**	**0.002**			**-**	**0.168**			**-**	**0.047**			**-**	**0.005**			**-**	**0.095**
balanced diet	**74 (86.05)**	**7 (50.00)**			**60 (85.71)**	**21 (70.00)**			**66 (85.71)**	**15 (65.22)**			**69 (85.19)**	**12 (63.16)**			**65 (84.42)**	**16 (69.57)**		
meat-based diet	**7 (8.14)**	**2 (14.29)**			**5 (7.14)**	**4 (13.33)**			**6 (7.79)**	**3 (13.04)**			**8 (9.88)**	**1 (5.26)**			**7 (9.09)**	**2 (8.70)**		
vegetarianism diet	**5 (5.81)**	**5 (35.71)**			**5 (7.14)**	**5 (16.67)**			**5 (6.49)**	**5 (21.74)**			**4 (4.94)**	**6 (31.58)**			**5 (6.49)**	**5 (21.74)**		
**Frequency ofcognitive activity**			**-**	**0.064**			**6.83**	**0.033**			**-**	**0.205**			**-**	**0.809**			**-**	**0.539**
never	**17 (19.77)**	**1 (7.14)**			**8 (11.43)**	**10 (33.33)**			**13 (16.88)**	**5 (21.74)**			**14 (17.28)**	**4 (21.05)**			**12 (15.58)**	**6 (26.09)**		
regular	**17 (19.77)**	**0 (0.00)**			**13 (18.57)**	**4 (13.33)**			**16 (20.78)**	**1 (4.35)**			**15 (18.52)**	**2 (10.53)**			**14 (18.18)**	**3 (13.04)**		
irregularly	**52 (60.47)**	**13 (92.86)**			**49 (70.00)**	**16 (53.33)**			**48 (62.34)**	**17 (73.91)**			**52 (64.20)**	**13 (68.42)**			**51 (66.23)**	**14 (60.87)**		
**SDSC**			**1.53**	**0.216**			**0.38**	**0.536**			**0.10**	**0.751**			**2.37**	**0.124**			**0.42**	**0.519**
normal	**52 (60.47)**	**6 (42.86)**			**42 (60.00)**	**16 (53.33)**			**44 (57.14)**	**14 (60.87)**			**44 (54.32)**	**14 (73.68)**			**46 (59.74)**	**12 (52.17)**		
anomaly	**34 (39.53)**	**8 (57.14)**			**28 (40.00)**	**14 (46.67)**			**33 (42.86)**	**9 (39.13)**			**37 (45.68)**	**5 (26.32)**			**31 (40.26)**	**11 (47.83)**		

Z: Mann-Whitney test, χ^2^: Chi-square test, -: Fisher exact

### 3.4 Multifactorial analysis of neurocognitive impairment in children with cancer

Stepwise binary logistic regression analyses were performed using the variables with *P*<0.10 in the univariate analysis as the independent variables and the below-normal categorical outcome of each dimension as the dependent variable (non-neurocognitive impairment = 0, neurocognitive impairment = 1).The results showed that disease duration, age, sex, radiotherapy status, father’s education level, dietary habit, and frequency of cognitive activity entered the regression model, with those variables having a final *P*<0.05 identified as independent influencing factors. These results are detailed in Table 5.

**Table 5:** Multifactorial logistic regression analysis.

Dimensions	Variables	*β*	*S*.*E*	*Z*	*P*	OR (95%CI)
Communication	**Father’s education level**					
	Primary school					1.00 (Reference)
	Junior High School	-0.87	1.03	-0.85	0.397	0.42 (0.06 ~ 3.14)
	High School	-1.17	1.12	-1.05	0.293	0.31 (0.03 ~ 2.76)
	University and above	-3.61	1.47	-2.46	**0.014**	0.03 (0.00 ~ 0.48)
	**Dietary habit**					
	balanced diet					1.00 (Reference)
	meat-based diet	1.65	1.08	1.53	0.127	5.22 (0.63 ~ 43.49)
	vegetarianism diet	2.55	0.99	2.58	**0.010**	12.84 (1.84 ~ 89.39)
	**Frequency of cognitive activity**					
	never					1.00 (Reference)
	regular	-14.39	2403.58	-0.01	0.995	0.00 (0.00 ~ Inf)
	irregularly	2.81	1.40	2.00	**0.045**	16.58 (1.06 ~ 258.93)
Gross motor	**Age**	-0.44	0.18	-2.38	**0.017**	0.65 (0.45 ~ 0.93)
	**Frequency of cognitive activity**					
	never					1.00 (Reference)
	regular	-1.12	0.77	-1.46	0.145	0.33 (0.07 ~ 1.47)
	irregularly	-1.21	0.57	-2.10	**0.035**	0.30 (0.10 ~ 0.92)
Fine motor	**Age**	-0.65	0.23	-2.80	**0.005**	0.52 (0.33 ~ 0.82)
	**Sex**					
	Male					1.00 (Reference)
	Female	-1.45	0.60	-2.42	**0.016**	0.23 (0.07 ~ 0.76)
	**Father’s education level**					
	Primary school					1.00 (Reference)
	Junior High School	-2.18	0.99	-2.20	**0.028**	0.11 (0.02 ~ 0.79)
	High School	-1.80	0.99	-1.81	0.071	0.17 (0.02 ~ 1.16)
	University and above	-2.74	1.01	-2.73	**0.006**	0.06 (0.01 ~ 0.46)
Problem Solving	**Age**	-0.71	0.24	-2.99	**0.003**	0.49 (0.31 ~ 0.78)
	**Eating**					
	balanced diet					1.00 (Reference)
	meat-based diet	-0.27	1.16	-0.23	0.816	0.76 (0.08 ~ 7.36)
	vegetarianism diet	2.27	0.81	2.79	**0.005**	9.69 (1.96 ~ 47.82)
Personal-Social	**Age**	-3.25	1.04	-3.12	**0.002**	0.04 (0.00 ~ 0.30)
	**Age group**					
	36–41 months					1.00 (Reference)
	42–47 months	0.08	1.48	0.05	0.956	1.08 (0.06 ~ 19.83)
	48–53 months	2.94	1.52	1.93	0.053	18.85 (0.96 ~ 369.52)
	54–59 months	4.17	1.93	2.16	**0.031**	64.45 (1.47 ~ 2833.96)
	≥60 months	7.30	2.91	2.51	**0.012**	1475.85 (4.92 ~ 442439.67)
	**Radiotherapy**					
	no					1.00 (Reference)
	yes	1.35	0.63	2.14	**0.032**	3.86 (1.12 ~ 13.29)

OR: Odds Ratio, CI: Confidence Interval

## 4 Discussion

In this study, we found that 49% of the children had abnormalities in at least one neurocognitive dimension, with the highest number of children (30%) showing abnormalities in gross motor dimension. This data highlighted the significant prevalence of cancer-related cognitive impairment (CRCI) in preschool children with cancer. The impact of CRCI on these children, whose expressive abilities are not yet fully mature is profound, and parents may not always be able to detect these changes in time. These findings revealed an important area of research and practice in pediatric oncology and neurocognitive function in pediatric patients.

Communication dimension was related to father’s educational level, dietary habit, and frequency of cognitive activity, which is similar to Cr’s [24] study that found that a higher educational level of the father significantly reduced the risk of children’s communication disorders. Especially in China, fathers often play the role of family economic supporters, and their higher education is usually accompanied by higher incomes, which not only creates superior socio-economic conditions for their children but also offers the possibility of accessing high-quality educational resources. In addition, a balanced and nutritious diet, is crucial for neurological function and has a positive impact on promoting communication skills. Regular participation in cognitive activity, on the other hand, further promotes neurocognitive ability through enhanced brain function exercises.

The main influencing factors on gross motor included child’s age and frequency of cognitive activity. Long-term treatment and limited opportunities for peer interaction often result in impaired gross motor function in children with disease. In addition, children who lack regular cognitive activity or have never participated in such activities show relatively low performance in gross motor.

Fine motor dimension was significantly related to age, sex, and father’s education level. The fine motor of younger children are usually not as mature as that of older children. It was also reflected gender differences in the performance of this ability, which is similar to the study of Gorp et al. [25], who pointed out that the higher prevalence of CRCI in female children may be attributed to the potential effects of estrogen on female brain structure and neurocognitive function, as described by Tan et al. [26]. In addition, Kesler et al. [27] revealed an association between caregivers’ educational background and children’s brain structure and neurocognitive performance: caregivers’ education was positively correlated with increased volume of white and gray matter, memory and processing speed, suggesting that caregivers’ education level indirectly contributed to the function of children’s fine motor function by, among other things, providing superior learning resources and environments.

Age and dietary habitwere associated with problem-solving function. Younger children experienced more severe neurocognitive impairment during treatment, which is consistent with the study of Rijmenams et al. [28]. Sleurs et al. [29] have further confirmed through a multicenter follow-up study involving 94 children with leukemia that the younger a child is diagnosis, the lower the subsequent IQ assessments tends to be. Developing communication skills, maintaining a good diet, and ensuring adequate supply of essential nutrients are crucial for normal brain function and problem-solving function. In addition, like communication skills, a good diet helps to provide necessary nutrients to support healthy brain function and enhance problem-solving skills.

Personal-social dimension were related to age and radiotherapy. Radiotherapy is a known risk factor for CRCI in children and may increase the risk of mild cognitineurocognitive impairment and dementia in adulthood [30]. Follin et al. [31] found in a cohort study that the neurocognitive function in children with leukemia who underwent radiotherapy was lower than that in the control group. Kim et al. [32] found that undergoing cranial radiotherapy was a significant risk factor for poor full-scale IQ performance in surviving children. Neurocognitive function may decrease in the years following radiotherapy [33], impacting children’s academic, life, and social adaptation, thereby affecting their socialization.

## 5 Conclusion

The study found that age, radiotherapy, sex, father’s education level and dietary habit were related to neurocognitive function in children with cancer. Therefore, we recommend that parents be aware of their important role in their child’s recovery, including guiding the child’s understanding of the illness, fostering neurocognitive development, and shaping the child’s psychological personality. Parents need to actively encourage their children to participant in cognitive activities, avoid prolonged use of electronic devices, and prevent prolonged social isolation, which are crucial for the neurocognitive development of preschool children. Additionally, we call on other researchers to explore psychosocial stress and investigate the specific biological mechanisms affecting preschool children in future studies, and we advise clinical staff to regularly assess the neurocognitive function of these children to implement early interventions.

## 6 Limitations

The limitations of this study included the absence of a healthy control group and the use of a cross-sectional research design, which compromised the study’s comprehensiveness. Besides. collecting data at a single time pointmade it unable to adequately explain the causal association between cancer-related neurocognitive impairment and numerous factors.
